# Molecular mechanisms underlying multi-level defense responses of horticultural crops to fungal pathogens

**DOI:** 10.1093/hr/uhac066

**Published:** 2022-03-14

**Authors:** Xiaodi Xu, Yong Chen, Boqiang Li, Zhanquan Zhang, Guozheng Qin, Tong Chen, Shiping Tian

**Affiliations:** 1 Key Laboratory of Plant Resources, Institute of Botany, The Innovative Academy of Seed Design, Chinese Academy of Sciences, Beijing 100093, China; 2 College of Life Sciences, University of Chinese Academy of Sciences, Beijing 100049, China

## Abstract

The horticultural industry helps to enrich and improve the human diet while contributing to growth of the agricultural economy. However, fungal diseases of horticultural crops frequently occur during pre- and postharvest periods, reducing yields and crop quality and causing huge economic losses and wasted food. Outcomes of fungal diseases depend on both horticultural plant defense responses and fungal pathogenicity. Plant defense responses are highly sophisticated and are generally divided into preformed and induced defense responses. Preformed defense responses include both physical barriers and phytochemicals, which are the first line of protection. Induced defense responses, which include innate immunity (pattern-triggered immunity and effector-triggered immunity), local defense responses, and systemic defense signaling, are triggered to counterstrike fungal pathogens. Therefore, to develop regulatory strategies for horticultural plant resistance, a comprehensive understanding of defense responses and their underlying mechanisms is critical. Recently, integrated multi-omics analyses, CRISPR-Cas9-based gene editing, high-throughput sequencing, and data mining have greatly contributed to identification and functional determination of novel phytochemicals, regulatory factors, and signaling molecules and their signaling pathways in plant resistance. In this review, research progress on defense responses of horticultural crops to fungal pathogens and novel regulatory strategies to regulate induction of plant resistance are summarized, and then the problems, challenges, and future research directions are examined.

## Introduction

Horticultural crops, including fruits, vegetables, and ornamentals, provide nutrients and biologically active substances as well as ornamental value [1]. Production and consumption of horticultural products are developing rapidly, thereby boosting agricultural economic growth, particularly in developing countries. However, there are many challenges and threats to production and processing of horticultural crops, among which fungal diseases are a primary cause of crop yield reductions, quality deterioration, and postharvest loss [1, 2]. Consequently, breeding of disease-resistant varieties and eco-friendly control of plant diseases caused by fungal pathogens have been the focus of research. Both approaches are largely based on the in-depth analysis of mechanisms underlying plant defense responses.

Plant defense responses to fungal pathogens are closely associated with plant developmental stages, fungal pathogenicity, and variable environmental conditions [3–6]. Plant defense responses are primarily categorized as preformed and induced. First, inherent physical structures and phytochemicals provide base defense against fungal pathogens [7, 8]. Second, defense signaling is activated after fungal pathogens are recognized, leading to induction of innate immunity, local defense responses, and systemic defense signaling. Innate pattern-triggered immunity (PTI) and effector-triggered immunity (ETI), with associated reprogramming of transcriptomes, proteomes, and metabolomes, are effective against fungal pathogens [3, 9, 10]. Local defense responses and systemic defense signaling are downstream responses in developing resistance. Local defense responses lead to localized hypersensitive response (HR) to prevent pathogen invasion, whereas systemic defense signaling primarily involves several phytohormone signaling pathways, resulting in systemic acquired resistance (SAR) and herbivore-induced resistance (HIR) [11–13].

In this review, recent progress in research on defense responses of horticultural crops to fungal pathogens and new regulatory strategies for induction of plant resistance are summarized. Then, limitations of previous studies are examined, and future research directions for genetic improvement in resistance of horticultural crops are proposed.

## Defense responses of horticultural crops to fungal pathogens

### Preformed defense responses

#### Physical barriers against fungal pathogen invasion

**Figure 1:**
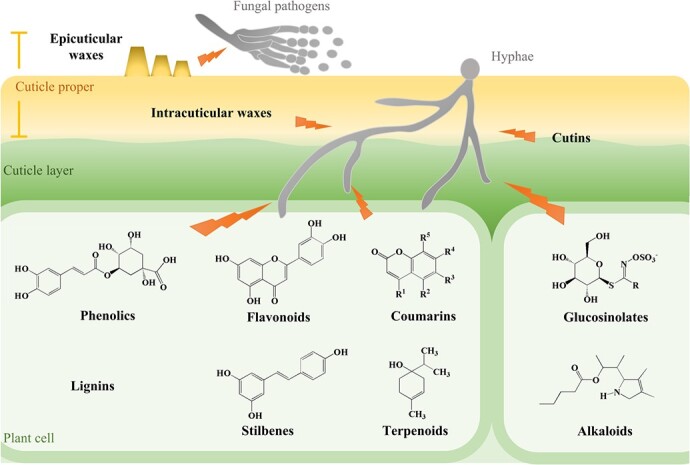
Physical barriers and phytochemicals involved in resistance of horticultural crops to fungal pathogens. Cuticles and cell walls are the outermost layers of plant cells and the first and important physical barriers against pathogen attack. Both the cuticle proper and inner cuticle layer are cutin-rich regions. Waxes of the cuticle proper are embedded in the cutins (intracuticular waxes) or cover the surface (epicuticular waxes). Accumulated cutins or waxes can increase disease resistance, and waxes themselves can be chemically antagonistic to fungal pathogens. Diverse antimicrobial phytochemicals induced by pathogens or accumulated after induction promote disease resistance. According to differences in their chemical structures, antimicrobial phytochemicals are categorized primarily as phenolics, flavonoids, coumarins, lignins, alkaloids, glucosinolates, terpenoids, and stilbenes. The chemical structures represent one example of the corresponding compound type, with side chains represented by R.

Cuticles and cell walls, the outermost layers of plant cells, are the first and important physical barriers against fungal pathogen invasion [7, 14] (Fig. 1). Plant cuticles are hydrophobic and therefore not conducive to spore germination of fungal pathogens [15]. Cuticles also maintain the mechanical strength necessary for normal plant growth. Many fungal species use their appressoria to penetrate the cuticular layer and then infect internal cells, whereas most necrotrophic fungi secrete cell wall-degrading enzymes to impair cuticles and facilitate infection [3, 16]. Plant cuticles are generally divided into two cutin-rich domains [17] (Fig. 1). The inner cuticle layer contains embedded polysaccharides, whereas the cuticle proper is rich in waxes that are embedded in cutins (intracuticular waxes) or cover the surface (epicuticular waxes) [18]. Cuticle composition and quantity are important factors that contribute to plant defense responses against pathogens. The *cutin deficient 1* (*cd1*), *cd2*, and *cd3* mutants of tomato (*Solanum lycopersicum*) fruit have less cutin accumulation, varied architectures, and elevated susceptibility to *Botrytis cinerea* [19]. Removal of cuticular wax from blueberry (*Vaccinium ashei*) fruit leads to disruption of reactive oxygen metabolism and impaired resistance during storage [20]. Similarly, removal of epicuticular waxes from cauliflower (*Brassica oleracea*) leaves facilitates attachment and penetration of fungal propagules during the early stages of *Alternaria brassicicola* infection [15]. Accumulation of cutins or waxes can increase resistance to disease. For example, the R2R3-MYB transcription factor (TF) MdMYB30 positively regulates wax biosynthesis in apple (*Malus domestica*) fruit by targeting *MdKCS1*, a homolog of the wax biosynthesis gene *KCS1* in *Arabidopsis*. The positive regulation leads to increased resistance against *Botryosphaeria dothidea* [21]. *CsWAX2* is a gene closely associated with wax and cutin biosynthesis in cucumber (*Cucumis sativus*); its overexpression or RNA interference (RNAi) lines exhibit increased or decreased susceptibility to *Botrytis cinerea*, respectively [22]. The *delayed fruit deterioration* (*dfd*) mutant of tomato fruit accumulates additional cutins, which delays softening and increases resistance to *B. cinerea* [23]. In addition, waxes themselves display chemical resistance to fungal pathogens. Waxes isolated from pear (*Pyrus bretschneideri*) fruit can inhibit spore germination and mycelial growth of *Alternaria alternata in vitro* [24]. A major component of cuticular wax from custard apple (*Annona squamosa*), 16-hentriacontanone (palmitone), also has antifungal activities [25]. Moreover, deposition of triterpenoids, major constituents of cuticular wax, is a major factor regulating nectarine (*Prunus persica*) fruit permeability and susceptibility to *Monilinia laxa* during fruit development [18]. Alterations in the cell wall also greatly affect defense responses [14]. Plant cell walls are primarily composed of cellulose and pectin, and pectin can be modified by pectate lyases (PLs) [26, 27]. In tomato fruit, RNAi of *PL* leads to increased cellulose and hemicellulose contents but decreased content of water-soluble pectin, as well as altered expression of some specific genes involved in hormone signaling, cell wall modification, oxidative stress, and pathogen resistance. All these variations result in reduced susceptibility to *B. cinerea* and a longer shelf-life [27].

#### Phytochemicals involved in defense responses

Phytochemicals with antimicrobial effects are important components of defense systems in plants [28]. Among such phytochemicals, phytoalexins are induced by external factors, whereas phytoanticipins occur naturally or increase after induction [28]. However, despite the conceptual distinction, the two types of compound are not fundamentally different. Antimicrobial phytochemicals are classified according to chemical structure and are primarily phenolics, flavonoids, coumarins, lignins, terpenoids, alkaloids, glucosinolates, and stilbenes [10, 29–32] (Fig. 1). Phenolics and flavonoids are secondary metabolites that constitute one of the most common and extensive groups of phytochemicals [33, 34]. These compounds inhibit pathogens by inducing membrane lipid peroxidation, which disrupts fungal cell membrane permeability and mitochondrial function [33, 35]. They can also induce defense responses via modulation of related metabolic or other signaling pathways. For example, among phenylpropanoid-derived phenolics and flavonoids, chlorogenic acid increases activities of defense-related enzymes and activates the salicylic acid (SA) signaling pathway to control postharvest decay of nectarine fruit caused by *Penicillium expansum* [36]. Luteolin induces phenylpropanoid metabolic activities at both transcript and protein levels in cherry (*Cerasus pseudocerasus*) fruit [37]. *p*-Coumaric acid (*p*-CA) and its methyl ester *p*-coumarate (MeCA) protect jujube (*Ziziphus jujuba*) fruit against *A. alternata* by activating antioxidant enzymes and regulating *PATHOGENESIS RELATED* (*PR*) genes and the phenylpropanoid pathway [38]. Similarly, terpenoids inhibit fungal growth and also induce disease resistance in strawberry (*Fragaria ananassa*) and orange (*Citrus sinensis*) fruits [39, 40]. Other phytochemicals also exhibit strong and stable broad-spectrum antifungal activity, suggesting they could be developed as alternatives to chemical fungicides against fungal diseases [32, 41].

In addition to these phytochemicals, phytohormones are also critical in regulating defense responses. SA, jasmonic acid (JA), and ethylene (ET) are the three primary defense hormones that regulate signaling networks of resistance against multiple pathogens [42, 43]. SA, JA, and their methyl esters are widely used in preharvest treatment and postharvest preservation to induce resistance in horticultural crops, including sweet cherry (*Prunus avium*) [44], loquat (*Eriobotrya japonica*) [45], tomato [46], and fresh-cut freesia flowers (*Freesia hybrida*) [47]. However, they have no direct antifungal activity on cultivated pathogens *in vitro* [47]. Other phytohormones, including abscisic acid (ABA) [48], brassinosteroids (BRs) [49], gibberellic acid (GA) [50], auxins (indole-3-acetic acid, IAA) [51], cytokinins (CKs) [52], and melatonin [53], either function independently or synergistically with other defense hormones in complex networks of immunity to protect horticultural crops.

### Induced defense responses

#### Innate immunity to counterstrike pathogens

**Figure 2:**
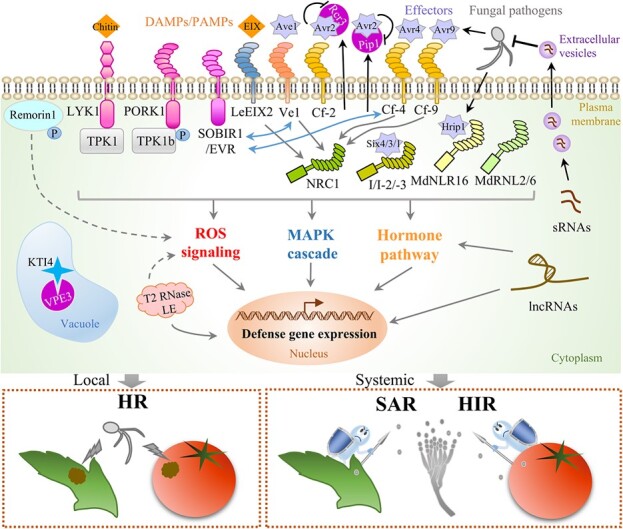
Recognition of fungal pathogens and induction of defense responses in horticultural crops. When fungal pathogens attack host plants, pathogen-associated molecular patterns (PAMPs) or plant-derived damage-associated molecular patterns (DAMPs) are recognized by specific pattern recognition receptors (PRRs), promoting pattern-triggered immunity (PTI), whereas fungal effectors are recognized by nucleotide-binding and leucine-rich repeat (NB-LRR or NLR) proteins and other resistance (R) proteins, triggering effector-triggered immunity (ETI). These processes result in effects on the MAPK cascade, reactive oxygen species (ROS) signaling, and hormonal crosstalk, which lead to further induction of local defense responses (HRs), systemic acquired resistance (SAR), and herbivore-induced resistance (HIR). Cysteine proteases (Rcr3, Pip1, and VEP3), ribonucleases (T2 RNases LE), and membrane proteins (remorin1) may be associated with HRs and contribute to disease resistance. Other novel components, such as long non-coding RNAs (lncRNAs) and small RNAs (sRNAs), also participate in defense responses against fungal pathogens. Gray arrows indicate immune responses, and black arrows indicate secretion. Bidirectional arrows indicate interactions. Dashed arrows indicate unconfirmed possibilities for the role of defense-related components. One arrow may indicate multiple steps. Lines with flat ends indicate inhibition. PORK1, PEPR1/2 ORTHOLOG RECEPTOR-LIKE KINASE1; TPK1b, TOMATO PROTEIN KINASE1b; LYK1, LysM RECEPTOR KINASE1; TPK1, TPK1b RELATED KINASE; SOBIR1/EVR, SUPPRESSOR OF BIR1-1/EVERSHED; LeEIX2, tomato resistance to ethylene-inducing xylanase (EIX); Ve1, resistance to *Verticillium dahlia*; Cf, resistance to *Cladosporium fulvum*; Ave1, avirulence on Ve1; Avr/Six, avirulence effector; NRC1, NB-LRR required for HR-associated cell death-1; I, resistance to *Fusarium oxysporum* f. sp. *lycopersici* (*Fol*); MdNLR16, *Malus domestica* NLR16; MdRNL, *M. domestica* NLR with a RESISTANCE TO POWDERY MILDEW8-like CC domain; Hrip1, hypersensitive response inducing protein 1; KTI4, Kunitz trypsin inhibitor 4.

When fungal pathogens penetrate physical barriers by modifying or degrading host cell walls, pattern recognition receptors (PRRs) may recognize conserved damage-associated molecular patterns (DAMPs) from plants or pathogen-associated molecular patterns (PAMPs) from pathogens and activate PTI [54]. Fungal pathogens can secrete effectors or virulence factors, which may be recognized by nucleotide-binding and leucine-rich repeat (NB-LRR or NLR) proteins and other resistance (R) proteins [55]. Such recognition may result in further ETI, which is postulated to be an accelerated and amplified PTI response [56]. These processes all lead to activation of defense genes, trade-offs between growth and defense, and defense responses (Fig. 2).

Plant PRRs are either receptor-like kinases (RLKs) or receptor-like proteins (RLPs) localized to the plasma membrane [54] (Fig. 2). RLKs have an ectodomain, a transmembrane region, and a cytoplasmic kinase domain, whereas the structure of RLPs is similar but without the cytoplasmic kinase domain [54, 57]. Chitin, polygalacturonases (PGs), and other PAMPs/DAMPs, are recognized by specific PRRs, leading to signaling cascades, including the MAPK cascade, Ca^2+^ influx, production of reactive oxygen species (ROS), and activation of defense genes [54, 55, 58]. Most PPRs involved in horticultural plant resistance to fungal pathogens have been identified in tomato. Highly similar to *Arabidopsis* PEPR1 and PEPR2 (two closely related RLKs), PEPR1/2 ORTHOLOG RECEPTOR-LIKE KINASE1 (PORK1) can phosphorylate TPK1b (TOMATO PROTEIN KINASE1b), a key regulator of tomato defense, and mediate systemin-induced resistance of tomato to *B. cinerea* [59]. Similarly, tomato LysM RECEPTOR KINASE (SlLYK1), an orthologue of the *Arabidopsis* chitin receptor, interacts with TPK1b RELATED KINASE (TRK1) [60]. SlLYK1 and TRK1 can regulate chitin-induced MPK6 phosphorylation, ROS accumulation, and expression of defense response genes that contribute to fungal resistance [60]. Moreover, tomato SUPPRESSOR OF BIR1-1/EVERSHED (SOBIR1/EVR), an ortholog of the *Arabidopsis* RLK, can interact with Ve1 and Cf-4 receptors and affect stability of those two RLPs. Thus, SOBIR1/EVR appears to function as a regulatory RLK for RLP-mediated resistance to *Verticillium dahliae* and *Cladosporium fulvum* [61]. The Ve1 receptor is an extracellular LRR (eLRR)-RLP that recognizes the Ave1 (for avirulence on Ve1) effector secreted by *V. dahlia*, as well as Ave1 homologs from *Fusarium oxysporum* f. sp. *lycopersici* (*Fol*) and *Cercospora beticola* [62]. The Cf resistance proteins (Cf-2, Cf-4, Hcr9-4E, Cf-5, Cf-9, and Hcr9-9B) are also eLRR-RLPs in tomato, but they confer specific recognition of some avirulence effectors (Avrs) secreted by *C. fulvum* [63]. With recognition, typical HR and resistance follow [63]. The RLP LeEIX2 in tomato detects the conserved PAMP ET-inducing xylanase (EIX), which induces an ROS burst, ET production, and the HR [64].

Owing to the increasing availability of plant genome sequences, NB-LRR genes, the largest class of *R* genes, have been generally identified in a genome-wide manner. Those large data sets are fundamental resources that can be mined for candidate genes against pathogens. NB-LRR genes are reported in diverse horticultural plants, including blueberry (*Vaccinium* spp.) [65], banana (*Musa acuminata*) [66], grapevine (*Vitis vinifera*) [67], kiwifruit (*Actinidia chinensis*) [68], hot pepper (*Capsicum annuum*) [66], tomato [69], potato (*Solanum tuberosum*) [70], yam (*Dioscorea rotundata*) [71], cabbage (*Brassica* spp.) [72, 73], cucumber [74], soybean (*Glycine max*) [75], and orchids (*Phalaenopsis equestris*, *Dendrobium catenatum*, *Gastrodia elata*, and *Apostasia shenzhenica*) [76]. A varied number of NB-LRR genes exhibit special evolutionary patterns among plant species. To date, only a few NB-LRR genes have been confirmed to function in response to fungal pathogens (Fig. 2). In tomato, NRC1 (NB-LRR required for HR-associated cell death-1), an NB-LRR protein, is required for the HR induced by RLPs (including LeEIX2, Cf-4, Cf-9, and Ve1) but also has intrinsic capacity to induce an HR and function upstream of the MAPK cascade [77, 78]. Moreover, the tomato NB-LRR genes *I*, *I-2*, and *I-3* confer resistance to *Fol* by recognizing the effectors Six4 (Avr1), Six3 (Avr2), and Six1 (Avr3), respectively [79]. Cabbage NB-LRR genes have diverse roles in responses against phytopathogens that depend on disease types and inoculation time [72, 73]. Two genes encoding NB-LRRs in cucumber, *CsRSF1* and *CsRSF2*, positively contribute to resistance against *Sphaerotheca fuliginea* by regulating expression of defense-related genes [80]. In addition, apple MdNLR16 interacts with the fungal effector hypersensitive response-inducing protein1 (Hrip1) to mediate resistance to *A. alternata*, in which sorbitol promotes transcriptional activation of *MdNLR16* by MdWRKY79 [81]. Two RNLs (NLRs with a RESISTANCE TO POWDERY MILDEW8-like CC domain) in apple, MdRNL2 and MdRNL6, interact to form heterodimeric complexes and provide broad-spectrum fungal resistance [82]. Further in-depth exploration of potential NB-LRRs and their mechanisms of action may substantially enrich the arsenal to counterstrike fungal pathogens.

#### Local defense responses to prevent pathogen invasion

To prevent further fungal pathogen invasion, plants have developed a series of responses that include the HR, cell wall modification, stomatal closure, callose deposition, phytoalexin production, and toxin degradation [38, 83, 84]. The HR is triggered upon the recognition of pathogens by host plants and is mediated primarily by avirulence genes of pathogens and *R* genes of hosts, often resulting in rapid cell death [85] (Fig. 2). The HR is an atypical and localized plant cell death, which is differentiated from other forms of programmed cell death (PCD) by some specific features, such as vacuolization and tonoplast rupture [86].

The HR is a highly controlled process that requires diverse proteases and regulatory components. However, these components remain largely unknown in horticultural plants. In the regulation of the HR in *Arabidopsis*, the roles of cysteine proteases, such as metacaspases, papain-like cysteine proteases (PLCPs), and vacuolar processing enzymes (VPEs), are well understood [86]. Several protease family members that respond to fungal pathogens have also been identified in horticultural plants (Fig. 2). Two paralogous PLCPs in tomato, Rcr3 (required for *Cladosporium* resistance3) and Pip1 (*Phytophthora* inhibited protease1), can be bound and inhibited by *Cladosporium*-secreted Avr2 [87], whereas recognition of the Avr2-Rcr3 complex results in HR [88]. Tomato fruits with reduced *SlVPE3* expression (*SlVPE3* RNAi lines) have increased susceptibility to *B. cinerea* [89]. Further quantitative proteomic and biochemical analyses showed that SlVPE3 is essential for the cleavage of the serine protease inhibitor KTI4 (Kunitz trypsin inhibitor4), which promotes tomato resistance to *B. cinerea*.

In addition to proteases, membrane proteins also appear to regulate cell death. Remorins are plant-specific proteins with multiple functions in growth, development, and responses to biotic and abiotic stresses [56, 90–93]. Overexpression of the *S. lycopersicum remorin1* (*SlREM1*) gene increases susceptibility of tomato to *B. cinerea*, whereas its heterologous expression triggers cell death in *Nicotiana benthamiana* leaves, depending on the phosphorylation of SlREM1 [94]. Three previously unreported proteins, blue copper protein-like (BCPL), cysteine-rich and transmembrane domain-containing protein A-like (CRTD), and nuclear cap-binding protein subunit2 (NCBP), are notably upregulated in *SlREM1*-expressing leaves and are associated with regulation of cell death. These findings indicate that SlREM1 positively regulates plant cell death and may be guarded by certain R proteins, although underlying mechanisms still require clarification. In addition, T2 ribonucleases (RNases) are RNA-degrading enzymes that also serve as cytotoxic agents, well known for their roles in cell death and defense responses [95]. Tomato T2 RNase *LE*-suppressed RNAi lines exhibit increased sensitivity to oxalic acid (OA, a cell death-inducing factor) and *B. cinerea*. The increase in sensitivity is associated with ROS accumulation and suppression of *pathogenesis-related* (*PR*) marker genes, including *PR1a*, *basic chitinase* (*chitinase*), *phenylalanine ammonia-lyase-1* (*PAL1*), and *1-aminocyclopropane-1-carboxylic acid synthase-2* (*ACS2*) [95]. Thus, additional research is needed on the direct targets of LE in the regulation of *PR* genes and other cellular processes in response to fungal pathogens.

#### Signaling involving long-distance transport of systemic molecules

**Figure 3:**
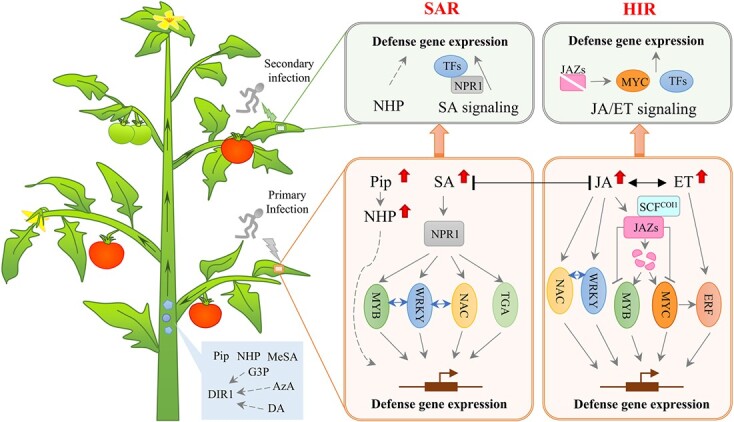
Molecular components and signaling pathways involved in systemic acquired resistance (SAR) and herbivore-induced resistance (HIR) triggered by elicitors. Fungal pathogen attack can increase endogenous contents of signaling molecules to trigger SAR or HIR. SAR is mediated mainly by salicylic acid (SA) signaling and to a lesser extent by *N*-hydroxypipecolic acid (NHP). NHP is produced from its precursor pipecolic acid (Pip), and both are mobile SAR signals that can accumulate at local and distal tissues in response to pathogens. SA signaling is activated at infected sites and thereby establishes SAR against secondary infection. The master regulator of SA and SAR is NPR1 (NONEXPRESSER OF PATHOGENESIS-RELATED GENES1). Transcription factors (TFs) are also involved in SA signaling to regulate defense genes. Jasmonic acid (JA) and ethylene (ET) pathways are essential for HIR. JA signaling antagonistically interacts with SA signaling. The JA receptor COI1 (CORONATIN INSENSITIVE1) forms an SCF^COI1^ complex and thereby targets jasmonate-ZIM domain (JAZ) repressors for degradation. Thus, related TFs are released to target other TFs or directly activate or repress transcription of JA-responsive genes. ET signaling synergistically interacts with JA signaling (as indicated by the black bidirectional arrow), and ERFs mainly function downstream of defense responses. Blue bidirectional arrows indicate interactions between TFs. Lines with flat ends indicate inhibition. Dashed arrows indicate unconfirmed possibilities for the role of mobile SAR signals. One arrow may indicate multiple steps. MeSA, methyl salicylic acid; AzA, azelaic acid; G3P, glycerol-3-phosphate; DA, abietane diterpenoid dehydroabietinal; DIR1, DEFECTIVE IN INDUCED RESISTANCE1.

After local defense responses are induced, systemic signaling may activate resistance in other adjacent tissues. Both PTI and ETI can trigger the production and long-distance transport of signaling molecules to induce SAR and HIR (Fig. 2). SAR is mediated primarily by SA signaling and to a lesser extent by *N*-hydroxypipecolic acid (NHP) [9]. In contrast to SAR, HIR is modulated by JA and ET [13]. Crosstalk among SA, JA, and ET, both synergistic and antagonistic, is common and crucial for defense responses against fungal pathogens [43] (Fig. 3).

*Systemic acquired resistance*. Elevated levels of SA in systemic tissues are a hallmark of SAR. Although SA is not considered a mobile SAR signal, its methyl ester MeSA is associated with long-distance communication and SA signaling is essential in establishing SAR to protect adjacent tissues from secondary infection [13]. When activated by SA, NONEXPRESSER OF PATHOGENESIS-RELATED GENES1 (NPR1) acts as a transcriptional coregulator of *PR* genes (marker genes for SAR) and is central to regulation of SAR development [13]. Moreover, the function of NPR1 is conserved across diverse species. Silencing of *GhNPR1* in *Gladiolus hybridus* leads to increased susceptibility to *Curvularia gladioli* [96]. In transgenic Indian mustard (*Brassica juncea*) lines overexpressing *BjNPR1*, activated expression of *PR* genes enhances resistance to *Alternaria brassicae* and *Erysiphe cruciferarum* [97]. Similarly, overexpression of *NPR1* in apple enhances resistance to *Venturia inaequalis*, *Gymnosporangium juniperi-virginianae*, and *Podosphaera leucotricha* [98, 99]. Overexpression of *VvNPR1.1* in grapevine also induces expression of *PR* genes and enhances resistance to powdery mildew [100]. In recent research, *NPR*, *PR*, and other defense-related gene families were identified in the grapevine genome. Notably, four *NPR* genes and seven *PR* genes are differentially expressed against powdery mildew infection [101]. Moreover, NPR1 interacts with several TGA TFs, leading to activation of *PR1* expression in *Arabidopsis* [102]. However, tomato NPR1 and TGA1.a are utilized by *B. cinerea* and *Alternaria solani* to suppress JA-responsive genes, *PROTEINASE INHIBITORS I* and *II* (*PI I* and *PI II*), thereby promoting lesion development [103].

TFs, such as WRKY, NAC, and MYB, form a complex regulatory network and also contribute to SA-induced resistance (Fig. 3). Expression of *WRKYs* can be induced by SA treatment in banana, orange, and apple [104–106]. Following SA treatment, the TFs MaWRKY1 and MaWRKY2 in banana fruit are activated and bind to promoters of *PR* genes, which contribute to resistance against *Colletotrichum musae* attack [104]. The TF CsWRKY70 can activate salicylate carboxymethyltransferase (*CsSAMT*) by binding to the W-box element in the *CsSAMT* promoter, implying involvement of WRKY in SA-induced resistance in orange against *Penicillium digitatum* [105]. The TF MdWRKY31 can be induced by the SA signal and then cooperates with hypersensitive-induced reaction (HIR) protein MdHIR4 to positively regulate apple resistance to *B. dothidea* [106]. Similar to the functions of WRKYs, MaNAC1, MaNAC2, and MaNAC5 in banana are induced upon *C. musae* infection and enhanced by exogenous SA treatment. Moreover, MaNAC5 can interact with MaWRKY1 and MaWRKY2 to regulate the transcription of *PR* genes in a cooperative manner [107]. Overexpression of the R2R3-MYB gene *MdMYB73* improves resistance of apple to *B. dothidea* with an increase in SA content and expression levels of SA-responsive genes [108]. In addition, interaction between MdMYB73 and MdWRKY3 further enhances resistance of apple against *B. dothidea* [108].

Several proteins and enzymes in several critical pathways are also involved in SA-induced resistance. These proteins, identified in postharvest sweet cherry and peach fruits, participate extensively in defense and stress responses (antioxidant proteins, heat shock proteins, and PR proteins), iron homeostasis (dehydrogenases), and the tricarboxylic acid cycle (triosephosphate isomerase, NADP-dependent malic enzyme, and NAD-dependent isocitrate dehydrogenase α subunit) [109, 110]. Notably, exogenous SA treatment stimulates transcription of peroxidase (POD) but limits that of catalase (CAT) in sweet cherry fruit [109]. By contrast, the combination of SA and *Pichia membranefaciens*, an antagonistic yeast commonly used for postharvest disease control, enhances the expression of CAT in peach fruit [110]. Further studies on proteins involved in stress responses, metabolism, and energy pathways will help illuminate the mechanisms of SAR.

The secondary metabolite NHP can also induce SAR [111] (Fig. 3). It is produced from its direct metabolic precursor, pipecolic acid (Pip), a mobile SAR signal, following a series of reactions mediated by AGD2-LIKE DEFENSE RESPONSE PROTEIN1 (ALD1), SAR-DEFICIENT4 (SARD4), and FLAVIN-DEPENDENT MONOOXYGENASE1 (FMO1) [111, 112]. During activation of SAR, Pip and NHP levels increase in pathogen-infected leaves and distal leaves [113]. Accumulation of Pip and NHP in adjacent tissues may orchestrate the accomplishment of systemic resistance [113]. In tomato, cucumber, and soybean, NHP can accumulate in response to pathogens [114]. In the absence of pathogen infection, heterologous expression of *Arabidopsis* genes necessary for NHP biosynthesis in tomato leaves also triggers SAR in distal tissues [115], confirming the possibility that bioengineering strategies can enhance NHP-induced resistance. Other metabolites associated with long-distance SAR signaling include azelaic acid (AzA), glycerol-3-phosphate (G3P), and abietane diterpenoid dehydroabietinal (DA), which have been identified primarily in *Arabidopsis* and tobacco [11, 116, 117]. To promote resistance activity, all three of these signals require the functional lipid transfer proteins DIR1 (DEFECTIVE IN INDUCED RESISTANCE1). Orthologs of *Arabidopsis* DIR1 have also been identified in horticultural plants, such as tomato, cucumber, and soybean, implying DIR1-mediated SAR signaling may be conserved across plant species [118].

*Herbivore-induced resistance*. JA/ET signaling is required predominantly for resistance against necrotrophic pathogens and herbivores [43]. Fungal pathogen attack and other elicitors greatly increase levels of endogenous JA and methyl jasmonate (MeJA) [46, 119], and JA signaling is crucial for HIR [120]. A core module of JA signaling in *Arabidopsis* contains three components: the F-box protein CORONATIN INSENSITIVE1 (COI1, jasmonate receptor), JASMONATE-ZIM DOMAIN (JAZ) proteins (transcriptional repressors), and a basic helix–loop–helix (bHLH)-type TF MYC2 [121–123]. The COI1 protein can form a functional SCF^COI1^ complex with Cullin1 and Skp1-like1, thereby targeting JAZ repressors for degradation and leading to activation or repression of MYC2-directed transcription of JA-responsive genes [124]. However, data on mechanisms of JA signaling in horticultural crops remain scarce. In common bean (*Phaseolus vulgaris*), *COI1* has been identified in response to white mold by a meta-quantitative trait locus (QTL) analysis, and therefore, it may be a potential target for marker-assisted selection (MAS) to obtain partial resistance to this fungal disease [125]. Overexpression of the grapevine gene *VvCOI1* in strawberry fruit induces the expression of defense genes, including *PPO* (polyphenol oxidase), *SOD* (superoxide dismutase), *POD*, *PAL*, *BG* (β-1,3-glucanase), and *chitinase*, and delays the *B. cinerea* infection process [120]. In susceptible tomato cultivars, COI1-dependent JA signaling enhances PCD induced by TA, a type of toxin secreted by *A. alternata* f. sp. *lycopersici*. This process may be attributed to interaction between JA signaling and ET and SA signaling, as well as the regulation of JA signaling on redox status and/or PCD components [126]. In addition, the bHLH TF MYC2 acts downstream of COI1 and orchestrates a hierarchical transcriptional cascade that regulates resistance of tomato to *B. cinerea* infection [121]. Modules MYC2-JA2L (JA2-Like) and MYC2-ERF.C3 (ET RESPONSE FACTOR.C3) positively regulate *THREONINE DEAMINASE* (*TD*) and *PR-STH2* [121]. In addition, MYC2 is involved in regulation of other JA-responsive genes in tomato, including *LEUCINE AMINOPEPTIDASE A* (*LAPA*), *PI-1*, *JAZ* genes, and JA biosynthetic genes such as *ALLENE OXIDE CYCLASE* (*AOC*), *ALLENE OXIDE SYNTHASE* (*AOS*), *OXOPHYTODIENOATE-REDUCTASE3* (*OPR3*), and *tomato LIPOXYGENASE D* (*TomLoxD*) [121].

In addition to MYC2, other TFs, such as WRKY, NAC, and MYB, play vital roles in JA-induced defense responses. Overexpression of *CsWRKY10* reduces the ROS level by affecting activities of antioxidant enzymes (SOD, CAT, and POD) and inhibits JA-mediated signaling but activates SA signaling, thereby enhancing resistance of cucumber to *B. cinerea* [127]. In banana fruit, MaNAC5 cooperates with MaWRKY1 and MaWRKY2 to transcriptionally regulate *PR* genes, which contributes to MeJA- and SA-induced resistance against *C. musae* [107]. In rose (*Rosa chinensis*), RcJAZ1, RcMYB84, and RcMYB123 physically interact with each other. With JA treatment, RcMYB84 and RcMYB123 are released following JAZ1 degradation to further activate defense responses against *B. cinerea* [128].

ET signaling is directly involved in defense responses and often interacts with the SA and JA pathways upon pathogen invasion [43] (Fig. 3). In a comparative transcriptomic analysis, high resistance to *P. expansum* at 0 to 48 h post-inoculation in apple is attributed to over-representation of genes associated with ET signaling, jasmonate signaling, and MYB TFs in the resistant genotype [129]. Akagi *et al*. [130] found that ET enhances the resistance of apple fruit to *B. cinerea* infection. ERFs mainly function downstream of ET signaling in stress responses, and further analysis showed that four *ERF* genes were induced after wounding or *B. cinerea* infection [130]. Similarly, 23 *RcERF* genes are induced in rose petals upon *B. cinerea* attack, among which *RcERF099* is a positive regulator in gray mold resistance [131]. Because they are strongly induced after infection, the genes *CpERF2* and *CpERF4* are associated with responses against pathogens in papaya (*Carica papaya*) fruit [132]. Moreover, MdERF11 positively regulates apple resistance to *B. dothidea* by promoting SA synthesis, suggesting a synergistic effect between ET and SA against *B. dothidea* [133].

## Novel strategies to activate defense responses in horticultural crops

In recent years, the overuse of traditional fungicides and antimicrobial agents has remained common because of frequent outbreaks of crop diseases. However, their use increases pathogen resistance to controlling agents and also threatens food safety and the environment [134]. Therefore, it is urgent that new strategies be developed for efficient disease control in order to meet requirements for sustainable development of the agricultural industry. The latest studies indicate that it is feasible and efficient to induce intrinsic resistance in horticultural crops via regulatory elements.

First, the discovery of cross-kingdom RNA trafficking has provided new prospects for crop protection. The necrotrophic fungus *B. cinerea* can produce small RNAs (sRNAs) as substantial effectors to suppress host immunity [135]. In turn, host plants introduce sRNAs into *B. cinerea* via extracellular vesicles that suppress the expression of genes associated with pathogenicity [136]. Overexpression or knockdown of transferred host sRNAs either promotes or reduces respective host resistance. Such results indicate that transferred host sRNAs contribute to host immunity by silencing fungal genes [136]. Moreover, the *B. cinerea dcl1 dcl2* double mutant, rather than *dcl1* or *dcl2* single mutants, shows lower virulence on various horticultural crops [137]. These results prompt the development of spray-induced gene silencing (SIGS) for eco-friendly crop protection. In recent work, environmental double-stranded RNA (dsRNA) could be taken up by many eukaryotic microbes with different efficiencies [138]. Moreover, topical application of dsRNA with high RNA uptake efficiency can markedly inhibit plant disease symptoms [138]. However, because the longevity of dsRNA is only approximately a week and the protection efficacy decreases over time, enhancement in sRNA stability is required before commercial use of SIGS.

In addition to sRNAs, long non-coding RNAs (lncRNAs) are important regulators in many biological processes, including in networks of plant defense responses [139] (Fig. 2). In tomato, lncRNA16397 induces *SlGRX* expression and further induces ROS accumulation and membrane injuries, whereas lncRNA33732 induces *RBOH* expression to increase H_2_O_2_ accumulation, thereby enhancing tomato resistance to *Phytophthora infestans* [140, 141]. In grapevine, 71 and 83 lncRNAs have been identified in response to powdery and downy mildew infections, respectively [142]. Further analysis suggests they modulate various responses, including signaling pathways of Ca^2+^, ROS, and phytohormones, cell wall reinforcement, PR protein expression, and secondary metabolism [142]. These findings enrich our understanding of lncRNA regulation of crop resistance in horticultural crops and also direct future efforts towards the regulation mechanisms of specific lncRNAs. Such efforts could help identify potential candidates for editing to improve broad-spectrum resistance to fungal pathogens in horticultural crops.

Translational control of mRNA via editing regulatory elements may be another efficient way to induce resistance in horticultural crops. Upstream open reading frames (uORFs) have widespread regulatory roles in modulating mRNA translation in eukaryotes [143]. In one interesting study, editing the uORF of *LsGGP2*, which encodes a key enzyme of ascorbic acid biosynthesis in lettuce (*Lactuca sativa*), increases oxidation stress tolerance and ascorbate content [144]. Moreover, transgene-free lines of plants with improved traits (such as *uorfLsGGP2* mutants) are readily obtained with CRISPR/Cas9, which has broader implications for crop improvement [144]. Additionally, with constitutive expression of *AtNPR1* in *35S*:*uorfs_TBF1_*-*AtNPR1* plants there is clear broad-spectrum disease resistance with relatively few adverse effects on growth [145]. Because uORFs are found extensively in eukaryotic mRNAs [146], it is highly possible that these regulatory elements could be manipulated to enhance broad-spectrum resistance with minimal adverse effects on normal growth and substantially benefit genetic improvement in horticultural crops.

Transcription activator-like effectors (TALEs), are a recent hotspot in executor gene discovery that can provide methods to regulate resistance. In *Xanthomonas*, TALEs can bind to effector binding elements (EBEs) in promoters of host plants and activate expression of host susceptibility (*S*) genes and *R* genes [147]. Tomato Bs4 (bacterial spot resistance locus no. 4) is a TALE-sensing NLR protein that recognizes AvrBs4 and triggers further host defense responses [148]. It is clear that additional efforts should be directed to the discovery of executor *R* genes in horticultural crops via TALE-based techniques. Over the past decade, several useful tools, such as TALE nuclease (TALEN) technology and the split-TALE (sTALE) system, have been established for genome editing, regulation of gene expression, and analysis of protein–protein interaction [149–151]. Development of these technologies will facilitate the improvement of broad-spectrum resistance to fungal pathogens in the breeding of horticultural crops.

## Conclusions

Because of the importance of fungal disease in pre- and postharvest loss of horticultural crops, researchers have focused on plant–pathogen interactions and control technology. Moreover, development and application of omics technologies have provided large data sets at multiple levels, which have further broadened insights into the defense responses against fungal pathogens.

Although great progress has been made in uncovering the mechanisms of defense responses in horticultural crops in the last two decades, problems remain to be resolved. Most characterized PRRs and NB-LRRs are reported in tomato as a model species, and only a few other horticultural species have been examined. Consequently, the specific functions of many of these receptors remain unclear, and how receptors recognize and transmit microbial signals still requires clarification. Because most postharvest pathogens are necrotrophic pathogens, further studies are required to determine how necrotrophic fungal effectors enter host cells and are then perceived by host plant cells. Answers to these questions may help increase understanding of plant–pathogen interaction as well as their coevolution. In addition, most of the in-depth knowledge on SAR and HIR is also derived from model plants, such as *Arabidopsis*, tobacco, and rice, whereas information on SAR and HIR in horticultural crops is scarce. In particular, commonalities and specificities of SAR and HIR in fruit, leaves, and roots as highly differentiated organs of horticultural crops need to be clarified. Because defense responses are complex and diverse, it is also urgent to untangle multiple crosstalk between different types of defense responses in horticultural crops, such as the components bridging PTI and ETI and coordination or antagonism between signaling of phytohormones. Moreover, genomic, transcriptomic, and epigenomic methods have identified many regulatory elements during the past several decades, including uORFs, sRNAs, lncRNAs, and other factors. Future research should be directed towards identifying specific regulatory mechanisms for resistance in horticultural crops and new targets to induce resistance.

Because multiple phytochemicals and microbial antagonists are currently known to activate plant resistance, broad-spectrum and long-term efficacy of the induction of resistance by such compounds should receive more attention, as well as commercialization of any alternatives to chemical fungicides. Furthermore, advances in genetic engineering during the past decade have considerably accelerated modifications to cuticular composition, cell walls, and other components involved in defense responses. When CRISPR/Cas9 technology has enabled gene editing at the level of a single base, further discovery of new allelic variants with beneficial consequences for crop traits related to defense responses will enrich genetic resources and greatly propel precise improvements in horticultural crops.

## Acknowledgements

This work was supported by projects from the National Natural Science Foundation of China (31930086, 32072637) and the Natural Science Foundation of Beijing Municipality (6212025). We sincerely apologize to colleagues whose works have not been cited because of space limitation.

## Author contributions

S.T. planned the review; X.X., Y.C., T.C., and S.T. wrote the manuscript. B.L., Z.Z., and G.Q. participated in discussion on the manuscript. All authors edited and reviewed the manuscript. 

## Conflict of interest statement

The authors declare no competing interests.
